# Evaluation of the Efficacy of a Vaccination Program against *Actinobacillus pleuropneumoniae* Based on Lung-Scoring at Slaughter

**DOI:** 10.3390/ani11102778

**Published:** 2021-09-23

**Authors:** Wolfgang Sipos, Vojislav Cvjetković, Branimir Dobrokes, Sabine Sipos

**Affiliations:** 1 Clinical Department for Farm Animals and Herd Management, University of Veterinary Medicine Vienna, 1210 Vienna, Austria; 2 Ceva Tiergesundheit GmbH, 40472 Düsseldorf, Germany; vojislav.cvjetkovic@ceva.com; 3 Poultry Vets GmbH, 2831 Warth, Austria; dbane28@gmail.com; 4 Veterinary Practice Schwertfegen, 3040 Neulengbach, Austria; s.sipos@tierarztpraxis-schwertfegen.at

**Keywords:** pneumonia, pleurisy, vaccination, *Mycoplasma hyopneumoniae*

## Abstract

**Simple Summary:**

*Actinobacillus pleuropneumoniae* is a serious infectious agent in pigs, inducing pleuropneumonias and often leading to death if left untreated. In order to document and quantify the effects of a specific vaccination campaign (Coglapix^®^) in a chronically diseased farrow-to-finish farm suffering from an acute episode in the fattening unit, lungs of 61 slaughter pigs in the mean in each run were evaluated using the electronic CLP^®^ lung score program three times between 2016 and 2020, with the last measurement during the acute episode before the start of specific vaccination, followed by two analyses, one 8 months after implementation of vaccination measures, and one five months after cessation of the vaccination program. Percentages of lungs affected by dorsocaudal pleurisy immediately before and during vaccination were 43 and 5, respectively. The APP-index decreased from 1.2 to 0.1. The incidence of bronchopneumonic lungs also decreased by 75%, showing that pathophysiologies of pneumonias and pleurisies are closely correlated. Remarkably, EP (enzootic pneumonia)-indices seemed to correlate better with the severity of clinical signs than APP-indices, especially when comparing the chronic phase of swine pleuropneumonia and the acute episode with peracute fatalities. In summary, vaccination measures against *Actinobacillus pleuropneumoniae* proved to be very effective in restoring lung health in the affected herd, which was underpinned by an increase in APP-related parameters after cessation of vaccination.

**Abstract:**

Porcine pleuropneumonia is of serious concern regarding lung health in pig production. Besides optimizing hygiene and pig management, specific vaccination against the causative agent, *Actinobacillus pleuropneumoniae*, is an important tool in the fight against this disease. As porcine pleuropneumonia may present with different clinical courses of disease, it is not always easy to objectively assess herd lung health state or to monitor improvements following specific therapeutic or prophylactic measures. Here, the effects of specific vaccination on lung health in a chronically diseased farrow-to-finish farm in Lower Austria experiencing an acute episode were monitored by means of an app-based electronic tool, enabling the scorers to document lung pathologies real-time at slaughter. At the time, when vaccination measures took effect, percentages of lungs affected by dorsocaudal pleurisy had decreased from 43 to 5 and the APP-index from 1.2 to 0.1, respectively. But not only pleurisies were diminished, also incidences and severities of bronchopneumonic alterations had dramatically decreased and exhibited interesting trends when set in connection to clinical signs. Overall, vaccination measures against *Actinobacillus pleuropneumoniae* proved to be very effective in restoring herd lung health.

## 1. Introduction

*Actinobacillus pleuropneumoniae* is one of the major etiological agents of severe lung lesions in pigs, leading to massive losses in pig industry, including in Austria, where this study was performed [1,2,3]. Several different clinical forms of *Actinobacillus pleuropneumoniae*-induced disease are known [3]. Acute and peracute forms present with dramatic changes in a pig’s behaviour and are often associated with severe dyspnoea, blood-tinged discharge through the mouth and nostrils, and high mortality. Subclinical and chronic forms are less prominent but go hand in hand with decreased rate of gain in body weight and are therefore also of economic importance. Endotoxins, glycosphingolipids, capsular polysaccharides, and RTX (repeats in toxin) toxins (ApxI-IV) have been identified as the most prominent virulence factors. Treatment and prevention of swine pleuropneumonia may be accomplished by antibiotics, such as ampicillin, amoxycillin, and cephalosporins, as well as by vaccination measures, respectively. The aim of this field intervention study was to evaluate the success of a specific vaccination program against *Actinobacillus pleuropneumoniae* in a chronically infected herd suffering from an acute episode by means of the lung scoring program CLP^®^ (Ceva Santé Animale, Libourne, France).

## 2. Materials and Methods

### 2.1. Farm History

The farm under investigation was a farrow-to-finish farm in Lower Austria with 65 sows and 400 fattening pigs, where surplus weaners are sold. Piglets are routinely vaccinated against *Mycoplasma hyopneumoniae* (Hyogen^®^, Ceva, Düsseldorf, Germany) and Porcine Circovirus Type 2 (PCV2; Ingelvac CircoFLEX^®^, Boehringer Ingelheim Vetmedica GmbH, Ingelheim am Rhein, Germany) in their 3rd week of life as well as Porcine Reproductive und Respiratory Syndrome Virus (PRRSV; Unistrain^®^ PRRS, Hipra, Amer, Spain) in their 5th week of life. Sows are routinely vaccinated against erysipelas and porcine parvovirosis as well as PRRS. Historically (recorded by our practice since 2016, but already present there earlier), livestock suffered from subclinical to chronic swine pleuropneumonia as diagnosed clinically and by means of bacteriological analysis of lung samples with two comparably mild acute episodes per year in the mean, presenting with 20–30% coughing animals, which were encountered with oral oxytetracycline treatments. Concomitantly, the lungs of fatteners were scored by means of CLP^®^ in October 2016 and March 2017.

**Figure 1:**
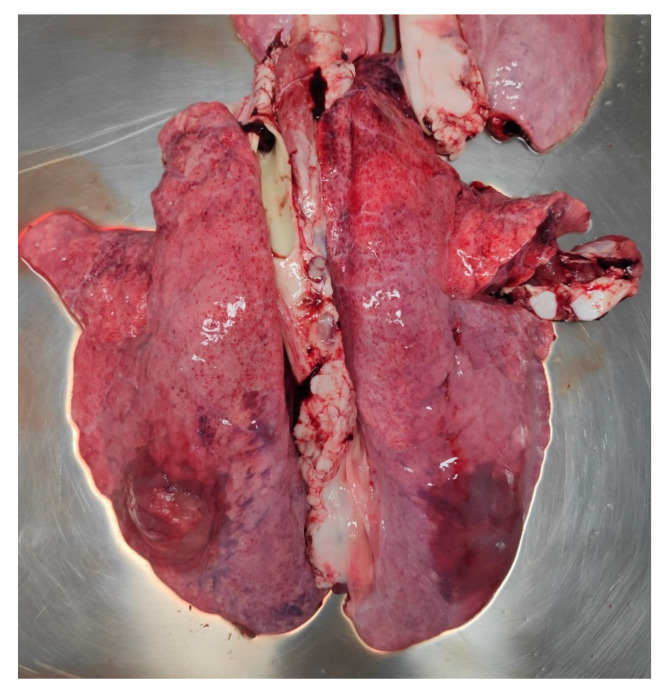
Pig, haemorrhagic pneumonia, sequester formation on left diaphragmatic lobe.

In June 2020, fatteners were affected by an acute episode without notable respiratory signs. Five pigs suffered from peracute pleuropneumonia and died within 24 hrs, showing the typical bloody-foamy nasal discharge. In general, feed uptake was depressed at herd level, leading to a three weeks extension of the fattening period of usually four months. Two of the perished 80 kg-pigs were autopsied by the farm veterinarian, who diagnosed haemorrhagic pneumonias being suggestive of an *Actinobacillus pleuropneumoniae* infection (Figure 1), and CLP^®^ lung scorings were also performed. As an immediate measure, oral amoxycillin treatment was started with good success, i.e., no further fatalities occurred. As the last bacteriological evidence for *Actinobacillus pleuropneumoniae* dated from 2016, also serum samples from 10 clinically ill animals were screened for ApxIV-specific antibodies by means of a commercial ELISA (IDEXX, Kornwestheim, Germany) and gave a positive result in 7 pigs and a suspicious one in another animal. Thus, swine pleuropneumonia was confirmed and a vaccination program using Coglapix^®^ (Ceva, Düsseldorf, Germany), a vaccine based on inactivated *Actinobacillus pleuropneumoniae* serotypes 1 and 2 as well as ApxI-III toxoids, was implemented two weeks later mid-June, i.e., when the per/acute episode was under control by means of antibiotic treatment. Pigs were vaccinated according to license in the 7th and 10th weeks of age. To evaluate the effect of the vaccination program on lung health, the CLP^®^ scoring procedure was again performed in March 2021, so that sufficient time had gone by since the implementation of the vaccination program. At that time, the farmer, being satisfied by the pleasant advancement of the herd health state but at the same time displeased with the costs of the vaccination program, had decided to stop it. Within four months, signs of subclinical to mild clinical signs, i.e., coughing, appeared again and culminated in three peracute fatalities within two weeks in August 2021. Another two pigs, one weaner and one fattener, which had been euthanized due to orthopaedic problems, exhibited pleuritic lesions at necropsy. To document the impact of cessation of the vaccination program, another batch of fatteners was then subjected to lung scoring. This time, judging the situation to be less dramatic than the first acute episode, the veterinarian started a new vaccination series immediately without antibiotic treatment. No further pigs succumbed, and the clinical situation improved again.

### 2.2. Lung Scoring

**Figure 2:**
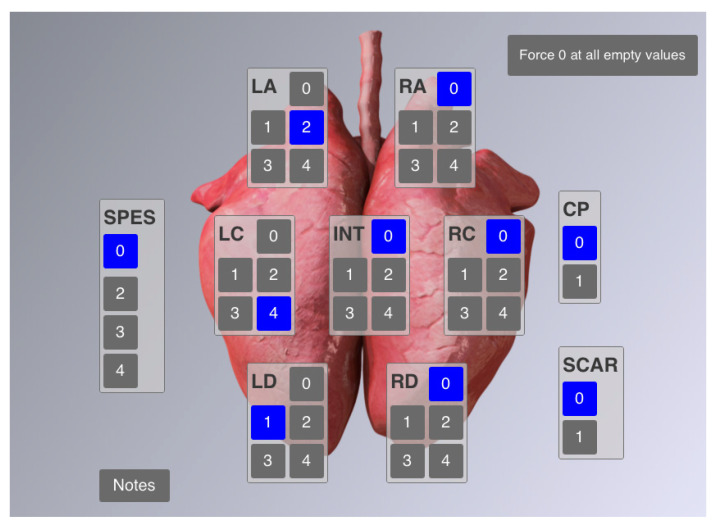
CLP^®^ user interface for entering lung scores. SPES: pleurisy values, CP: cranial pleurisy (i.e., SPES value = 1). Lobe-associated fields display the values according to the modified Madec-Kobisch-Score, indicating enzootic pneumonia-like lesions.

All lung scorings were performed by the same scorer using CLP^®^, which is an electronic tool that can be real-time operated on a tablet at the slaughter line (Figure 2) [4]. Particularly, the CLP^®^ categorizes (I) lung lesions according to a modified Madec-Kobisch-Score and a delineated EP (enzootic pneumonia)-score, thus considering lesions predominantly caused by *Mycoplasma hyopneumoniae*, with a theoretically possible range of values between 0 and 28 and (II) dorsocaudal pleurisies, which are judged by means of a SPES (Slaughterhouse Pleurisy Evaluation System)-value and a delineated APP-index within a possible range between 0 and 4 [5,6,7]. Undisputedly, not all dorsocaudal pleurisies are caused by *Actinobacillus pleuropneumoniae*, but may also be a consequence of infection by other pathogens such as *Glaesserella parasuis* and various mycoplasmas. In the field, however, swine pleuropneumonia-like lesions are strongly correlated with *Actinobacillus pleuropneumoniae* [3,8].

## 3. Results and Discussion

In 43% of the pigs screened before onset of vaccination, i.e., in June 2020, lungs were affected by dorsocaudal pleurisies (DP). The APP-index was 1.2 and thus slightly lower than index values of former scorings in 2016 and 2017. In vaccinated animals, however, the percentage of DP-affected lungs had decreased dramatically to 5 and the APP-index to 0.1. After cessation of vaccination measures, APP-index raised again to 0.8. Scoring results over the total observation period (2016–2021) are presented in Table 1.

APP-indices before vaccination have to be considered as very high when compared to those measured at slaughterhouse checks from fatteners in Austria and Germany in a survey including more than 36.000 lungs ranging regionally between 0.4 and 0.7 [9]. In general, the comparison of pre- and post-vaccination data shows a distinct improvement of lung score values and thus lung health. Remarkably, not only the APP-index decreased by 98% as a consequence of vaccination against *Actinobacillus pleuropneumoniae*, but also the percentage of bronchopneumonic lungs had been reduced to 25% and the one of dorsocaudal pleurisy to 12% of the initial pre-vaccination values. The fact that *Mycoplasma hyopneumoniae*-associated score values also improved dramatically in consequence of the *Actinobacillus pleuropneumoniae*-specific vaccination measure is due to the reciprocal dependency of score parameters, as has been shown already before [10]. These observations underline the close interdependency of pathoanatomical and thus also pathophysiological processes within PRDC (porcine respiratory disease complex), the multifactorial respiratory disease syndrome in pigs [11], and demonstrate that pneumonias and pleurisies should always be regarded as closely related pathological entities in the porcine respiratory tract from the physician‘s point of view.

Another interesting point is the observation that lung scores do not seem to closely reflect the clinical situation in the case of swine pleuropneumonia. During the subacute/chronic phase, APP-indices were higher than during the acute/peracute phase. In this case it seems, that pleurisy’s intensities may have slightly ameliorated as time passed. Thus, the frequency of fibrinous pleurisies was lower by trend when the acute episode of 2020 occurred, but nevertheless, from an instinctual point of view, one would rather expect higher APP-index values in cases of more severe clinical signs, as were evident during the acute episode with peracute fatalities. Consequently, neither APP-index values reflecting the degree of dorsocaudal pleurisies nor the physical examination outcomes alone seem to permit an estimation of disease severity, especially during subclinical or chronic periods, albeit lung scoring presents as the more reliable method for that purpose. Interestingly, EP-indices seemed to more closely reflect the clinical situation, as the EP-index was highest during the acute episode of 2020. EP-indices give an impression on the severity of bronchopneumonias and low chronic/subclinical-phase levels of approximately 2 have to be judged as “expectable” in comparison with published data of *Mycoplasma hyopneumoniae*-vaccinated fatteners in a case-control study, whereas the EP-index value exceeding 3 during the acute episode is clearly indicating a more severe clinical situation, with a median of 5 being typical of EP-diseased, unvaccinated animals in Austria [10]. The apparently counter-intuitive relation of APP-index and clinical presentation in combination with EP-index values shows that systemic illness as provoked by pneumonia leads to more serious clinical pictures when compared to pleurisy alone. Another point worth mentioning is that DP incidences and APP-index values were closely correlated and thus had nearly parallel curve courses. This means that a higher incidence of pleuritic lungs might be associated with a higher severity of pleurisies.

CLP^®^ data not only allow self-evaluation of the veterinarian, but also enable him/her to convince the farmer of the rationale of implemented vaccination measures. In the presented case, the acute pleuropneumonia episode could be attributed to omitting the weaners’ PRRS-vaccination by the farmer in the course of emotional pressure due to the COVID-19 pandemic, resulting in an increased PRRSV-associated impact on the animals‘ immune system, which in consequence could no longer cope with *Actinobacillus pleuropneumoniae*. This is good evidence for the clinical significance of many viral pathogens, primarily PRRSV and PCV2, due to their immunosuppressive actions [12,13] in the context of PRDC and the resulting need for management measures to stabilize herd health [10,11].

After the farmer had decided to stop vaccination measures against *Actinobacillus pleuropneumoniae* again due to economic considerations, it took only 4–5 months for the clinical picture of the herd to change again. However, this time, fatalities were clearly more infrequent in comparison to the preceding acute episode before implementation of the specific vaccination. This was reflected by the CLP^®^ data with pleurisy-related values having raised, albeit not to the level seen during the first, more severe acute episode, and with no major changes of bronchopneumonic incidences and EP-index value. Seemingly, previous vaccination against *Actinobacillus pleuropneumoniae* had supported the animals to develop a stronger immune response following vaccination against *Mycoplasma hyopneumoniae*, which was still active.

In conclusion, app-based real-time lung scoring at the slaughter-line combined with integrated statistical analysis allows to gain an immediate differentiated overview on herd lung health and is thus a valuable tool for quantifying the effects of therapeutic interventions. Here, it was used for proving the efficacy of a combined whole cell/toxoid vaccine against *Actinobacillus pleuropneumoniae* in restoring lung health at herd level. The flare-up of the pleuropneumonia after cessation of the vaccination measures emphasizes the necessity of strict adherence to metaphylactic vaccination management in case of endemic pathogens until they have been eradicated, if this is the aim. With regard to the interpretation of lung score parameters, not only absolute index values, but also the dynamics of single parameters and the interplay between these should be monitored. In this study, elevated (and raising, respectively) APP-indices were indicative of a clinical event related to *Actinobacillus pleuropneumoniae*-infection on herd level, but the severity of the disease was strongly affected by EP-index levels.

## 4. Conclusions

CLP^®^ lung score data of a case of porcine pleuropneumonia are presented and the dynamics during endemic disease with an acute episode, followed by the installation of a specific vaccination program has been discussed. Attention was turned to the score data, sometimes seemingly not entirely matching clinical observations. When considering pathophysiological connections, these ambiguities are resolved. Concerning APP, CLP^®^ data proved a valuable means of monitoring herd lung health, especially during chronic disease, as peracute and acute courses of the disease are much more evident clinically. In addition, the success of a specific vaccination was clearly documented by use of CLP^®^.

## Figures and Tables

**Table 1:** Overview on lung score data using CLP^®^ in a pig herd suffering from chronic swine pleuropneumonia and a per/acute outbreak in 2020, followed by implementation of a specific vaccination program against *Actinobacillus pleuropneumoniae* and a control scoring in 2021, followed by a score after stopping vaccination again.

Parameter	October 2016 *n =* 68	March 2017 *n* = 89	July 2020 *n* = 30	March 2021 *n* = 56	August 2021 *n* = 60
History	subacute/chronic disease		acute episode	vaccinated animals	acute episode
Bronchopneumonia (%)	69	63	43	11	5
EP-index	2.7	1.6	3.2	0.2	0.1
Scars (%)	22	40	10	0	0
Cranial pleurisy (%)	54	48	20	13	37
Dorsocaudal pleurisy (%)	50	60	43	5	30
APP-index	1.4	1.6	1.2	0.1	0.8

## References

[1] Elicker S., Scherer N., Sipos W. (2009). Retrospective analysis of the aetiology of respiratory disease in weaned piglets in Austria. Tierärztl. Umsch..

[2] Elicker S., Mayrhofer E., Scherer N., Fischer L., Weissenböck H., Sipos W. (2009). Retrospective analysis of the aetiology of respiratory diseases of Austrian fattening pigs as well as gilts and sows. Wien. Tierarztl. Monat..

[3] Gottschalk M., Taylor D.J., Straw B.E., Zimmerman J.J., D’Allaire S., Taylor D.J. (2006). Actinobacillus pleuropneumoniae. Diseases of Swine.

[4] Cvjetković V., Sipos S., Szabo I., Sipos W. (2018). Clinical efficacy of two vaccination strategies against *Mycoplasma hyopneumoniae* in a pig herd suffering from respiratory disease. Porc. Health Manag..

[5] Madec F., Kobisch M. (1982). Bilan lésionnel des poumons de porcs charcutiers à l‘abattoir. Journées Rech. Porc. Fr..

[6] Ostanello F., Dottori M., Gusmara C., Leotti G., Sala V. (2007). Pneumonia disease assessment using a slaughterhouse lung-scoring method. J. Vet. Med. A.

[7] Dottori M., Nigrelli A.D., Bonilauri P., Merialdi G., Gozio S., Cominotti F. (2007). Proposal for a new grading system for pleuritis at slaughterhouse. The S.P.E.S. (slaughterhouse Pleuritis evaluation system) grid. Large Anim. Rev..

[8] Merialdi G., Dottori M., Bonilauri P., Luppi A., Gozio S., Pozzi P., Spaggiari B., Martelli P. (2012). Survey of pleuritis and pulmonary lesions in pigs at abattoir with a focus on the extent of the condition and herd risk factors. Vet. J..

[9] Waehner C., Cvjetković V., Antonczyk C., Krejci R. Survey of lung lesions of pigs at slaughter with the Ceva Lung Program in Germany and Austria. Proceedings of the 12th European Symposium of Porcine Health Management.

[10] Sipos W., Dobrokes B., Meppiel L., Sailer J., Friedmann U., Cvjetković V. (2020). Association of lung score findings from slaughter pigs with their vaccination status against *Mycoplasma hyopneumoniae* and PCV2. Berl. Münch. Tierärztl. Wochenschr..

[11] Ruggeri J., Salogni C., Giovanni S., Vitale N., Boniotti M.B., Corradi A., Pozzi P., Pasquali P., Alborali G.L. (2020). Association between infectious agents and lesions in post-weaned piglets and fattening heavy pigs with porcine respiratory disease complex (PRDC). Front. Vet. Sci..

[12] Sipos W., Duvigneau C., Pietschmann P., Holler K., Hartl R., Wahl K., Steinborn R., Gemeiner M., Willheim M., Schmoll F. (2003). Parameters of humoral and cellular immunity following vaccination of pigs with a European modified-live strain of porcine reproductive and respiratory syndrome virus (PRRSV). Viral Immunol..

[13] Kekarainen T., Segalés J. (2015). Porcine circovirus 2 immunology and viral evolution. Porc. Health Manag..

